# TG68, a Novel Thyroid Hormone Receptor-β Agonist for the Treatment of NAFLD

**DOI:** 10.3390/ijms222313105

**Published:** 2021-12-03

**Authors:** Andrea Caddeo, Marta Anna Kowalik, Marina Serra, Massimiliano Runfola, Andrea Bacci, Simona Rapposelli, Amedeo Columbano, Andrea Perra

**Affiliations:** 1 Unit of Oncology and Molecular Pathology, Department of Biomedical Sciences, University of Cagliari, 09042 Monserrato, Italy; andreamarco.caddeo@unica.it (A.C.); ma.kowalik@unica.it (M.A.K.); marina.serra@unica.it (M.S.); 2 Department of Pharmacy, University of Pisa, 56126 Pisa, Italy; massimiliano.runfola@farm.unipi.it (M.R.); andrea.bacci@phd.unipi.it (A.B.); simona.rapposelli@unipi.it (S.R.)

**Keywords:** thyromimetics, steatosis, triglycerides, Resmetirom, NASH, MAFLD

## Abstract

Activation of thyroid hormone receptor β (THRβ) has shown beneficial effects on metabolic alterations, including non-alcoholic fatty liver disease (NAFLD). Here, we investigated the effect of TG68, a novel THRβ agonist, on fatty liver accumulation and liver injury in mice fed a high-fat diet (HFD). C57BL/6 mice fed HFD for 17 or 18 weeks, a time when all mice developed massive steatohepatitis, were then given TG68 at a dose of 9.35 or 2.8 mg/kg for 2 or 3 weeks, respectively. As a reference compound, the same treatment was adopted using equimolar doses of MGL-3196, a selective THRβ agonist currently in clinical phase III. The results showed that treatment with TG68 led to a reduction in liver weight, hepatic steatosis, serum transaminases, and circulating triglycerides. qRT-PCR analyses demonstrated activation of THRβ, as confirmed by increased *mRNA* levels of *Deiodinase-1* and *Malic* *enzyme-1*, and changes in lipid metabolism, as revealed by increased expression of *Acyl-CoA Oxidase-1* and *Carnitine palmitoyltransferase-1*. The present results showed that this novel THRβ agonist exerts an anti-steatogenic effect coupled with amelioration of liver injury in the absence of extra-hepatic side effects, suggesting that TG68 may represent a useful tool for the treatment of NAFLD.

## 1. Introduction

Non-alcoholic fatty liver disease (NAFLD), recently renamed metabolic associated fatty liver disease (MAFLD) [1], is a major cause of liver-related morbidity and mortality affecting one third of the global population worldwide [1,2,3]. NAFLD covers a wide spectrum of pathological conditions, ranging from simple hepatic steatosis, also known as non-alcoholic fatty liver (NAFL), to the more severe form, namely non-alcoholic steatohepatitis (NASH) [4]. About 59% of the patients bearing NAFLD develop NASH that can further progress to cirrhosis, ultimately leading to hepatocellular carcinoma (HCC) [5,6,7,8]. Although NAFLD can occur also in lean subjects, it is strictly related to insulin resistance, obesity, and other metabolic alterations, and it is considered the hepatic manifestation of metabolic syndrome. No pharmacological therapies are approved for the treatment of this syndrome, although some experimental drugs are currently in later stages of clinical development [9].

Thyroid hormones (THs) are essential regulatory molecules for normal growth, development, and for maintaining metabolic homeostasis [10]. Most of the activities elicited by THs are mediated by nuclear thyroid hormone receptors (THRs), which are members of the nuclear hormone receptor family and act as ligand-activated transcription factors [11]. There are two main THR isoforms encoded by separated genes, namely THRα and THRβ, the expression of which widely varies amongst tissues. THRβ is the most abundant isoform in the liver and mediates the effects of THs on hepatic carbohydrate and lipid metabolism [12,13,14]. Conversely, experimental and clinical evidence suggests that alterations of the cellular TH signaling in the liver play a key role in the onset and progression of several liver-associated diseases, such as NAFLD and HCC [15,16,17]. The use of THs as therapeutic agents has been hampered for a long time by the lack of selectivity and the consequent harmful adverse side effects on several organs and apparatus, such as heart and musculoskeletal system. Since these effects are mediated mainly by the THRα, the selective activation of THRβ is an appropriate method to develop new pharmacological treatments with a reduced side effect profile for several chronic liver diseases. During the past two decades, a promising therapeutic strategy for liver diseases derived from THRβ1-selective thyromimetics, such as GC-1 (Sobetirome), KB2115 (Eprotirome), and the Hep-Direct prodrug VK2809 (MB07811), which have reproduced 3,5,3′-triiodothyronine (T3)-related biological effects without overt cardiotoxic effects [18,19]. Nevertheless, their possible clinical application was interrupted at the same stage of the clinical experimentation. Despite these disappointing results, it has been reported that Resmetirom (also known as MGL-3196), a liver-directed THRβ agonist orally administered, entered a phase 3 clinical trial. In a 36-week clinical study in patients with NASH, MGL-3196 treatment led to a significant reduction in liver triacylglycerides (TAGs), circulating LDL cholesterol and TAGs, and to a higher rate of NASH resolution compared to placebo [20]. Moreover, Harrison et al. reported that treatment with MGL-3196 reduced markers of fibrosis in adults with biopsy-confirmed NASH. Thus, MGL-3196 has been proposed for the treatment of the full spectrum of NAFLD and associated dyslipidemias [21]. Parallel to MGL-3196 other liver-selective T3 analogues binding THRβ have been developed in the last few years. Recently, our group reported the synthesis of a novel halogen free THRβ-selective agonist IS25 and its pro-drug TG68, using GC-1 as a scaffold compound [22]. Both IS25 and TG68, when tested for cytotoxicity and ADME-Tox/off-target liability in vitro, revealed a convincing lack of toxicity and the capacity to reduce total lipid accumulation into lipid droplets with an effect comparable, or even higher, than equimolar doses of T3 [22]. Further in vivo studies showed that both compounds were able to increase hepatocyte proliferation, in a way quite similar to the thyroid hormone T3, but in the absence of liver damage, cardiotoxicity, and renal hypertrophy [23], demonstrating a marked hepato-specificity. Based on these encouraging preliminary results, we decided to investigate prodrug TG68 in a NASH mouse model in order to explore its potential to treat this liver syndrome.

Here, we report that TG68 strongly reduced hepatic fat accumulation and liver injury in mice fed a high-fat diet (HFD) in the absence of overt deleterious effects in extra-hepatic tissues, such as kidney or heart. Notably, while most of the effects of TG68 were almost identical to those of MGL-3196, circulating triacylglycerides (TAGs) were significantly reduced only by TG68.

## 2. Results

### 2.1. A High Dose of TG68 Caused a Reduction in Liver Volume and Hepatic Steatosis

**Figure 1:**
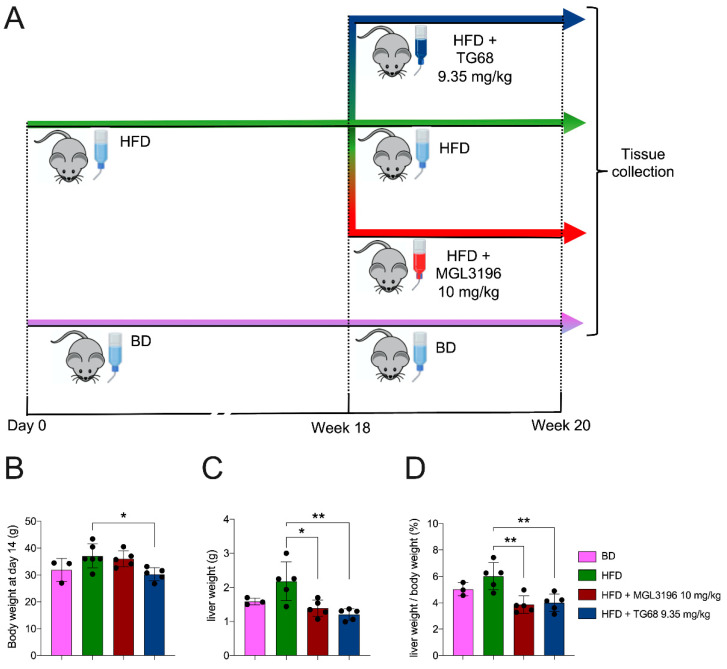
**Effect of two-week treatment with TG68 or MGL-3196 on liver weight.** (**A**) Experimental design and timeline of the in vivo experiments; (**B**) body weight; (**C**) liver weight; (**D**) liver weight/body weight ratio. Groups were compared using unpaired two-tailed Student’s *t*-test and non-parametric Mann–Whitney U Test, or one-way ANOVA followed by Tukey *post hoc* analysis. Values represent mean ± standard deviation (*n* ≥ 3). * *p* < 0.05, ** *p* < 0.01. Abbreviations: BD, basal rodent diet; HFD, high fat diet.

Previous studies demonstrated that MGL-3196 could be safely administered for up to 23 days at the dose of 10 mg/kg/day [24] to diet-induced obese mice. For this reason, in the first set of our experiments, we administered TG68 at the equimolar dose of MGL-3196 to mice fed a HFD for 18 weeks. After having confirmed the presence of hepatic steatosis, mice were randomized into experimental groups: BD (basal diet), HFD, HFD+MGL-3196 (10 mg/kg), and HFD+TG68 (9.35 mg/kg). All the compounds were given in drinking water for two weeks (Figure 1A). The treatment with TG68 caused only a slight effect on body weight compared to HFD and HFD+MGL-3196 groups (Figure 1B). On the other hand, both compounds caused a significant and comparable reduction in liver weight and liver weight/body weight ratio compared to HFD-fed untreated rats (Figure 1C,D).

**Figure 2:**
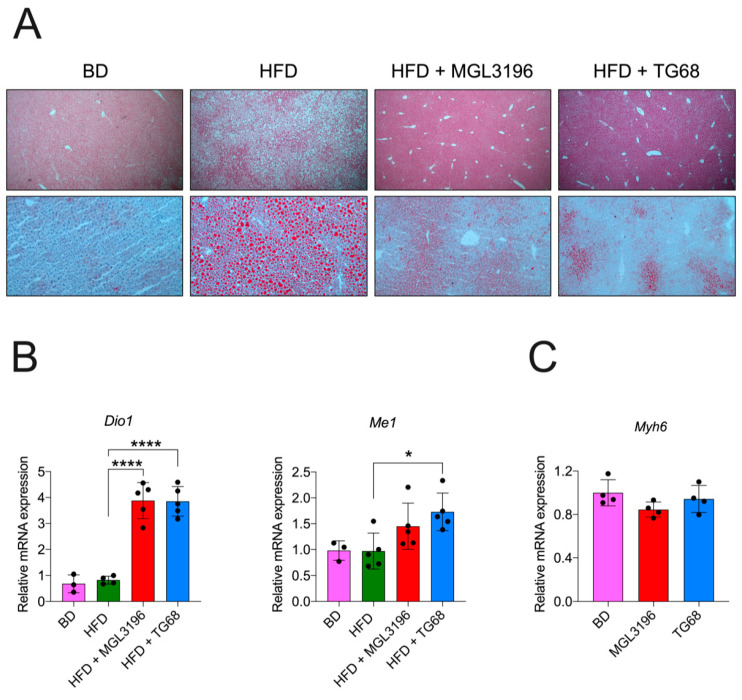
**Two-week treatment with MGL-3196 or TG68 specifically reduced hepatic neutral fat accumulation.** (**A**) Representative images of liver sections stained with hematoxylin and eosin (5×, upper panel) or Oil Red O (10×, lower panel) at 10 mg/kg (MGL-3196) or 9.35 mg/kg (TG68); (**B**) gene expression analysis of hepatic *Dio1*, *Me1* and (**C**) cardiac *Myh6*. All measurements were normalized to *β-actin.* Groups were compared using unpaired two-tailed Student’s *t*-test and non-parametric Mann–Whitney U Test, or one-way ANOVA followed by Tukey *post hoc* analysis. Values represent mean ± standard deviation. * *p* < 0.05, **** *p* < 0.0001. Abbreviations: BD, basal rodent diet; HFD, high fat diet.

To determine whether the observed reduction in liver weight was due to an amelioration of the hepatic steatosis, liver samples from both groups were subjected to comparative pathological analysis. The microscopic analysis of hematoxylin and eosin (H&E) stained sections confirmed the presence of stage 4 steatosis in all the HFD livers from untreated mice, whilst treatment with the two thyromimetics was associated with an impressive reduction in the fat content (Figure 2A), and amelioration of classical signs of cell damage typically associated with NAFLD, such as cell swelling, Mallory–Denk bodies, acidophilic bodies, or spotty necrosis. The Oil Red O (ORO) staining for neutral lipid content supported the histological observation (Figure 2A).

To confirm that the observed effects were associated with the activation of THRs, the expression of two target genes were investigated. As shown in Figure 2B, both compounds increased the expression of *Type 1 Iodothyronine deiodinase* (*Dio1*) and *Malic enzyme 1* (*Me1*). To corroborate the functional THRβ selectivity of TG68 at a dose of 9.35 mg/kg for two weeks, we also investigated the cardiac gene expression of the *Myosin heavy chain 6* (*Myh6*), a gene under the control of the THRα isoform. qRT-PCR analysis did not show any increase in the cardiac expression of *Myh6* in mice treated with TG68 or MGL-3196 (Figure 2C), thus confirming the in vivo specificity for the THRβ.

Altogether, these data demonstrated that two weeks of treatment with a high dose of MGL-3196 or TG68 specifically reduced the neutral fat content in the liver without any histological sign of cardiotoxicity.

### 2.2. The Strong Reduction in Intrahepatic Neutral Lipid Accumulation of HFD-Fed Mice Is Achieved Also by a Lower Dosage of TG68

Since the treatment of HFD-fed mice with 9.35 mg/kg of TG68 for two weeks led to a significant reduction in liver steatosis, further experiments were conducted to determine whether even a lower dose of TG68 administered for a longer time could lead to the same beneficial effects.

**Figure 3:**
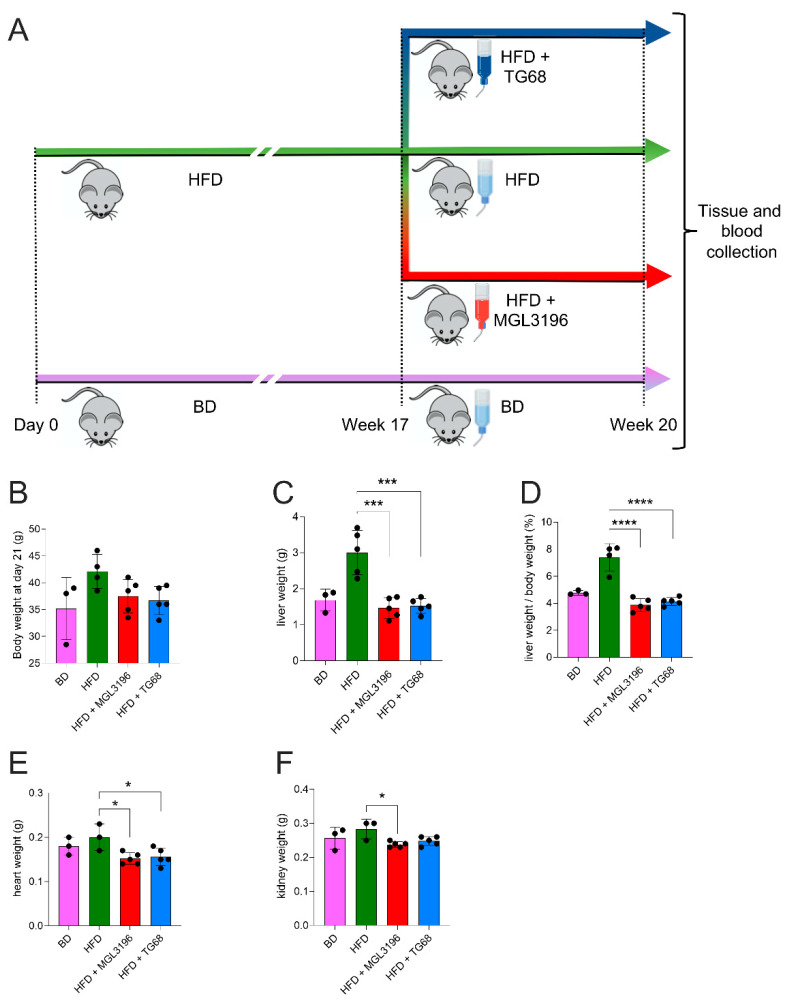
**Three-week treatment with TG68 (2.8 mg/kg) or MGL-3196 (3 mg/kg) reduced body and liver weight.** (**A**) Experimental design and timeline of the in vivo experiments. (**B**) Body weight B; (**C**) liver weight; (**D**) liver weight/body weight ratio; (**E**) heart weight; (**F**) kidney weight. Groups were compared using unpaired two-tailed Student’s *t*-test and non-parametric Mann–Whitney U Test, or one-way ANOVA followed by Tukey *post hoc* analysis. Values are shown as mean ± standard deviation (*n* ≥ 3). * *p* < 0.05, *** *p* < 0.001, **** *p* < 0.0001. Abbreviations: BD, basal rodent diet; HFD, high fat diet.

To this aim, mice fed HFD for 20 weeks were given TG68 or MGL-3196 at a dose of 2.8 or 3 mg/kg, respectively, in drinking water for the last three weeks (Figure 3A). This treatment caused a slight reduction in body weight compared to animals fed HFD alone (Figure 3B), although it was not statistically significant. Meanwhile, as expected, HFD-mice displayed a massive increase in liver weight compared to mice fed BD; administration of TG68 significantly reduced the liver weight as well as the liver weight/body weight ratio. Similar results were obtained with MGL-3196 (Figure 3C,D). No changes on heart or kidney weights were observed following administration of both drugs, suggesting their safety in in vivo conditions (Figure 3E,F).

**Figure 4:**
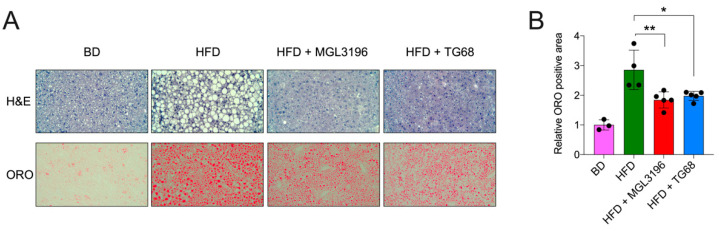
**Three-week treatment with TG68 or MGL-3196 reduced hepatic neutral fat accumulation.** (**A**) Representative images of liver sections stained with H&E (20×, upper) or Oil Red O (10 ×, lower); (**B**) Oil Red O staining positive area quantification by using ImageJ. Data were normalized to controls (BD). Groups were compared using unpaired two-tailed Student’s *t*-test and non-parametric Mann–Whitney U Test, or one-way ANOVA followed by Tukey *post hoc* analysis. Values are shown as mean ± standard deviation. * *p* < 0.05, ** *p* < 0.01. Abbreviations: BD, basal rodent diet; HFD, high fat diet; H&E, hematoxylin and eosin; ORO, Oil Red O.

According to the results shown in Figure 4A, H&E-stained liver sections revealed the presence of a macrovesicular steatosis in the liver of HFD-fed mice, whereas only a negligible number of round pale vesicles was seen in the liver of mice treated with TG68 or MGL-3196. This finding was supported by ORO staining (Figure 4A). Quantitative analysis performed on ORO-stained sections revealed a 30% reduction in the liver fat content in mice given TG68, compared to HFD-fed mice (Figure 4B). Altogether, these data demonstrated that the oral treatment with a lower dose of TG68 was safe, and it significantly reduced the accumulation of TAGs in the liver. As shown in Figure 4A,B, the efficacy of TG68 proved to be similar to that of MGL-3196.

### 2.3. TG68 Reduced Liver Damage and the Levels of Circulating Triacylglycerides

**Figure 5:**
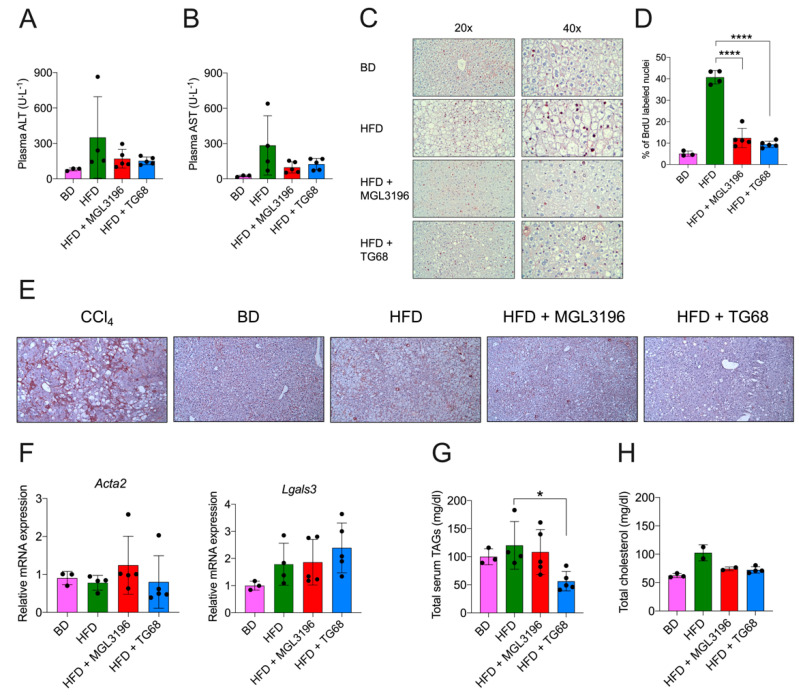
**Three-week treatment with TG68 reduced liver damage, hepatocyte proliferation, and serum triacylglycerides and cholesterol levels.** (**A**,**B**) Effect of TG68 and MGL-3196 on serum alanine aminotransferases (ALT) and aspartate aminotransferases (AST); (**C**) representative images of liver sections immunostained for BrdU detection, (**D**) BrdU Labeling Index. Labeling index was calculated as the number of BrdU-positive hepatocyte nuclei/per field at 40× magnification. Additionally, 50 fields per rat liver were scored; (**E**) representative images of liver sections immunostained for alpha-smooth-muscle actin (α-SMA) detection (20×). Mice treated with carbon tetrachloride (CCl_4_) were used as positive controls; (**F**) gene expression analysis of *Actin alpha 2* (*Acta2*) and *Galectin 3* (*Lgals3*) in mice liver. All measurements were normalized to *β-actin;* (**G**,**H**) effect of TG68 and MGL-3196 on serum triacylglycerides and cholesterol levels. Groups were compared using unpaired two-tailed Student’s *t*-test and non-parametric Mann–Whitney U Test, or one-way ANOVA followed by Tukey *post hoc* analysis. Values are shown as mean ± standard deviation. * *p* < 0.05, **** p < 0.0001. Abbreviations: BD, basal rodent diet; HFD, high fat diet; AST, aspartate aminotransferases; ALT, alanine aminotransferases; BrdU, 5-Bromo-2′-deoxyuridine; CCl_4_, carbon tetrachloride; TAGs, triacylglycerides.

Since the pathological analysis indicated an improvement in liver histology, to further explore whether the anti-steatogenic effect of TG68 also resulted in a reduction in hepatocyte damage, we analyzed the levels of serum transaminases. As shown in Figure 5A,B, TG68 caused a strong decrease, albeit not statistically significant, in plasma alanine aminotransferases (ALT) and aspartate aminotransferases (AST) compared to mice fed HFD alone. Similar results were obtained with MGL-3196. Since cell injury associated with NAFLD leads to cell death, compensatory hepatocyte proliferation is an indirect index of the severity of liver damage. To this aim, liver sections were immunostained to detect 5-Bromo-2′-deoxyuridine (BrdU) incorporation in the newly-synthesized DNA. As expected, the percentage of BrdU-positive nuclei in mice fed HFD and exposed to TG68 or MGL-3196 was significantly lower than that of mice fed HFD alone (Figure 5C,D). This finding is even more interesting when we consider that thyromimetics have been shown to be mitogenic for hepatocytes [25].

Since NAFLD progression is associated with fibrogenesis, the expression of alpha-smooth muscle actin (α-SMA) was investigated. However, histological analysis as well as immunohistochemical staining for α-SMA did not display any sign of hepatic fibrosis (Figure 5E), showing that 20 weeks of exposure to HFD are not sufficient to induce fibrogenesis. As expected, treatment with the thyromimetic had no effects on α-SMA immuno-staining. Furthermore, qRT-PCR analysis did not show any significant change in the expression of *Actin alpha 2* (*Acta2*) or *Galectin 3* (*Lgals3*), genes previously associated with fibrosis progression (Figure 5F) [26,27,28].

Notably, mice treated with TG68, but not MGL-3196, showed a significant reduction in the levels of serum triacylglycerides (TAGs) (Figure 5G) pointing out the potential role of TG68 for the treatment of dyslipidemia. A trend towards a reduction in circulating cholesterol was also observed with both the thyromimetics, although the difference with those from mice fed HFD alone was not statistically significant (Figure 5H).

### 2.4. TG68 Specifically Induces Thyroid Hormone Receptor Target Genes

**Figure 6:**
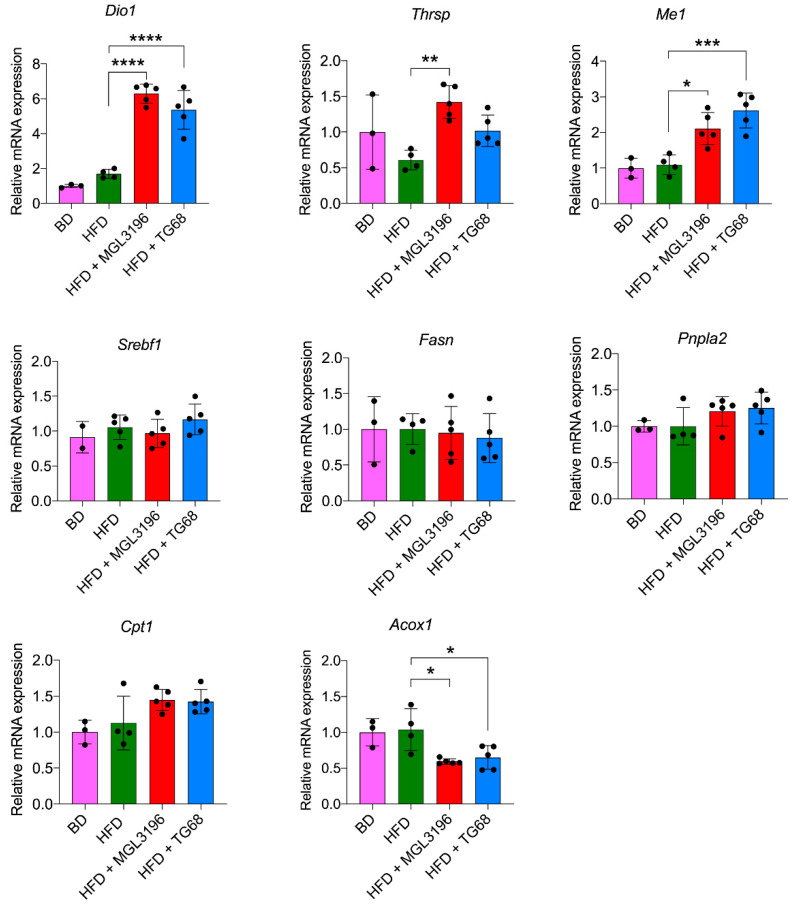
**Gene expression analysis of hepatic genes involved in thyroid hormone, lipid, and glucose metabolism following a three-week treatment with TG68 or MGL-3196.** Total hepatic RNA was extracted from snap-frozen tissues using Qiazol reagent and reverse-transcribed using high-capacity cDNA reverse transcription kit. All measurements were normalized to *β-actin*. Groups were compared using unpaired two-tailed Student’s *t*-test and non-parametric Mann–Whitney U Test, or one-way ANOVA followed by Tukey *post hoc* analysis. Values are shown as mean ± standard deviation (*n* ≥ 3). * *p* < 0.05, ** *p* < 0.01, *** *p* < 0.001, **** *p* < 0.0001. Abbreviations: *Acta2*, *Actin alpha 2*; *Acox1*, *Acyl-CoA Oxidase-1*; BD, basal rodent diet; *Cpt1*, *Carnitine palmitoyltransferase-1*; *Dio1*, *Deiodinase-1*; *Fasn*, *Fatty acid synthase*; HFD, high fat diet; *Lgals3*, *Galectin 3*; *Me1*, *Malic enzyme-1*; *Myh6*, *Myosin heavy chain 6*; *Pnpla2*, *Patatin-like phospholipase domain containing 2*; *Srebf1*, *Sterol regulatory element binding transcription factor 1*; *Thrsp, Thyroid hormone responsive*.

To assess whether the anti-steatogenic effect of a low dose of TG68 was mediated by activation of THRs, we assessed by qRT-PCR the mRNA levels of THR-target genes, such as *Dio1*, *Thyroid hormone responsive* (*Thrsp*), and *Me1*. As shown in Figure 6, while HFD did not affect the expression of these genes, both compounds caused increased expression of all the investigated genes. The extent of induction caused by TG68 was comparable to that of MGL-3196.

Based on the profound effect of TG68 on the reduction in hepatic lipid accumulation, we investigated the expression of several genes involved in *de novo* lipogenesis, such as *sterol regulatory element binding transcription factor 1* (*Srebf1*), *fatty acid synthase* (*Fasn*), and *patatin-like phospholipase domain containing 2* (*Pnpla2*). However, no statistically significant differences in the mRNA levels of these genes were observed following treatment with either TG68 or MGL-3196 (Figure 6).

On the other hand, a significant downregulation of *Acyl-CoA Oxidase-1* (*Acox1*) and an overexpression of *Carnitine Palmitoyltransferase-1* (*Cpt1*) were observed in the livers of mice treated with TG68 or MGL-3196, highlighting their important role in improving mitochondrial fatty acid oxidation and ROS metabolism in mice subjected to HFD [29,30].

Taken together, these data suggest that TG68 activates hepatic THRβ ameliorating the hepatic fat content by acting on the expression of genes involved in mitochondrial fatty acid beta-oxidation.

### 2.5. TG68 Showed Liver Specificity

**Figure 7:**
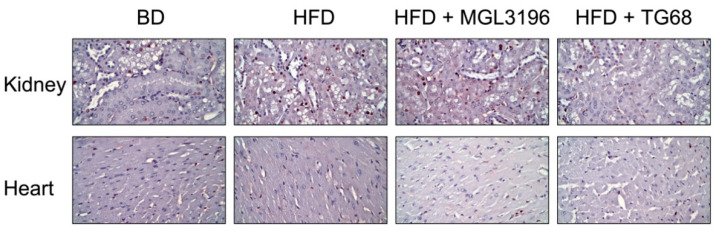
**Three-week treatment with TG68 or MGL-3196 did not cause injury to extra-hepatic tissues.** Representative images of kidney and heart sections immunostained for BrdU detection (20×). Abbreviations: BD, basal rodent diet; HFD, high fat diet.

While T3 exerts several beneficial effects on hepatic metabolism, its potential therapeutic use is impaired by the several side effects on other organs (i.e., kidney and heart) exerted through the activation of THRα [31]. To exclude extrahepatic effects of TG68, we investigated whether the treatment with this drug affected the heart and the kidney. As previously shown (Figure 3E,F), low doses of TG68 administered for three weeks did not cause any significant change in the heart or kidney weight. To better explore whether TG68 had toxic effects on extra-hepatic organs, we performed a pathological analysis on heart and kidney slides. Neither thyromimetics caused microscopic signs of organ damage or significant changes in the BrdU immunostaining (Figure 7). These results suggested that, while a three week-treatment with TG68 promotes the removal of lipids from the liver and protects from hepatic injury, it does not cause discernible damage to extra-hepatic organs/tissues.

## 3. Discussion

The present study addressed the evaluation of the recently synthesized THRβ agonist TG68 on hepatic fat accumulation and liver injury in mice fed HFD, a model closely recapitulating the complex pathological events associated with NAFLD in humans. It also examined the potential impact of TG68 on extra-hepatic tissues, such as kidney and heart, known target organs of the thyroid hormone T3.

As to the first point, the results demonstrated that TG68 robustly reduced neutral fat accumulation and ameliorated liver cell injury. Interestingly, the efficacy of TG68 was comparable to that of MGL-3196, another THRβ agonist that has already entered a phase III clinical trial [21]. Another interesting and novel observation is that the strong reduction in steato-hepatitis caused by TG68 was reached in the context of a very short time exposure (2 to 3 weeks), thus ensuring the possibility of treating patients with multiple cycles therapy, rather than with continuous treatment. Indeed, NAFLD is a chronic disease whose resolution may require long-term treatment.

qRT-PCR analyses suggested that the anti-steatogenic effect could be associated with the expression of genes involved in mitochondrial fatty acid beta-oxidation, while the *mRNA* levels of most of the genes involved in lipid metabolism were unaffected.

As already observed in previous studies [22,23], the administration of TG68 in HFD-fed mice lowered the serum levels of ALT and AST, revealing that, in addition to revert a condition of simple steatosis, it may also efficiently ameliorate the background of cell death typical of NASH. Furthermore, TG68 can also significantly reduce serum TAGs. Notably, the effect on circulating TAGs levels represents the main difference observed in this study when comparing the effects of TG68 with those of MGL-3196. NASH is frequently associated with metabolic co-morbidities such as obesity, elevated TAGs and cholesterol, diabetes, and systemic hypertension, which are well-known features of the pathological setting referred to as metabolic syndrome [32,33]. Therefore, the strong reduction in TAGs (30%) caused by TG68 indicates its possible contribution in the reduction in the risk of cardiovascular events associated with liver diseases. Interestingly, the significant reduction in serum TAGs induced by TG68 suggested it could also be able to regularize the lipid metabolism in subjects with multifactorial dyslipidemia but without metabolic syndrome.

Another relevant finding of this work is the lack of toxicity of TG68 on extra-hepatic organs, such as heart and kidney. Indeed, while one of the most frequent side effects hampering the clinical use of T3 is cardiotoxicity, neither macroscopic nor histological analyses of this organ showed detectable signs of toxicity after three weeks of treatment. Although hyperthyroidism is associated with heart failure at the time of diagnosis in 6% of cases, in vivo studies suggested that myocardial pathological remodeling does not appear before several weeks of hyperthyroidism [34]. Since modifications of gene expression precede morphological changes, the expression of *Myh6*, a typical target of activated THRα, was investigated in HFD-fed mice treated with TG68. The lack of effects of TG68 on *Myh6* added further support to the specificity of TG68 for THRβ and, consequently, to the safety of this novel thyromimetic.

Increasing evidence suggests that liver hypothyroidism can be, in part, responsible for NASH, and that the incidence of hypothyroidism is higher in patients with NAFLD/NASH relative to age-matched controls [16,35]. Not surprisingly, THRβ agonists have been shown to provide beneficial effects by mimicking thyroid hormone in its ability to reduce hepatic TAGs and atherogenic lipids, such as lipoproteins, in the absence of unwanted toxic effects, mainly mediated by THRα. Although most of the literature indicates hypothyroidism as a risk factor for NAFLD, there are some case reports linking hyperthyroidism to liver injury [36,37]. Aside from Graves’ disease, in which liver involvement (including cholestasis) can be seen as part of autoimmune dysregulation, thyrotoxicosis remains a rare cause of direct liver function alteration. Our results demonstrated that chronic administration of TG68 (up to 3 weeks) was not only not associated with an increase in transaminases but resulted in normalization of both ALT and AST levels in a context of increased HFD-induced oxidative stress. Therefore, we believe this new drug is sufficiently safe for use in long-term therapies. Nevertheless, more preclinical studies are needed prior to its therapeutic use.

Based on our current knowledge of NASH and on the lack of approved pharmacological therapies, the present study strongly suggests that TG68 represents an innovative hepatospecific THRβ agonist with no detectable toxicity, and, consequently, an attractive candidate for the treatment of NASH.

## 4. Materials and Methods

### 4.1. Mice and Drug Treatments

Four-week-old C57BL/6 male mice were purchased from Charles River Italy (Calco, Italy). The mice were housed for two weeks at 22 °C with free access to basal rodent diet (Mucedola s.r.l., Settimo Milanese, Italy) and drinking water with a 12 h light/dark daily cycle before starting the experiments. Two experimental protocols were adopted.

Experimental Protocol 1. Sixteen mice were fed ad libitum a high fat diet (HFD, 42% kcal/fat diet, Mucedola s.r.l., Settimo Milanese, Italy) containing sucrose and 1.25% cholesterol for 18 weeks. Animals were then split in three groups: group 1 (*n* = 6) was maintained on HFD for further two weeks; group 2 (*n* = 5) was fed a HFD plus MGL-3196 (10 mg⋅kg^−1^ in drinking water, MedChemExpress, Monmouth Junction, NJ, USA) for two weeks; group 3 (*n* = 5) was fed a HFD plus TG68 (9.8 mg⋅kg^−1^, in drinking water) for two weeks.

Experimental Protocol 2. Fourteen mice were fed ad libitum a high fat diet (HFD) for 17 weeks and then split into three groups: group 1 (*n* = 4) was maintained on HFD for further three weeks; group 2 (*n* = 5) was fed a HFD plus MGL-3196 (3 mg⋅kg^−1^ in drinking water, MedChemExpress,) for three weeks; group 3 (*n* = 5) was fed a HFD plus TG68 (2.8 mg⋅kg^−1^, in drinking water) for three weeks. The doses of 3 mg⋅kg^−1^ and 9.8 mg⋅kg^−1^ were selected based on the dose–response to MGL-3196 on cholesterol lowering in DIO mice [24]. Notably, Kannt et al. [38] calculated that, for mice with a body weight of about 35–38 g, this dose corresponds to a human equivalent dose of about 19 mg for an 80 kg person, which is four times lower than the dose of 80 mg used in NASH patients [20,38]. Equimolecular doses of TG68 were utilized in order to compare the effects of the two drugs. An additional control group of mice (*n* = 3) received basal diet (Mucedola srl) all throughout the experimental time. All animals were sacrificed under isoflurane anaesthesia twenty weeks after the beginning of HFD or basal diet administration. Blood and tissues, including liver, intestine, heart, and kidney, were collected (for experimental protocol see Figure 1A and Figure 3A).

All animal procedures were performed according to procedures approved by the Ethic Commission of the University of Cagliari. Animal experiments and procedures were approved by the local Ethical committee and by the Italian Ministry of Health (the ethical code is N.1247/15-PR), complied with national ethical guidelines for animal experimentation, and were conducted in accordance with the guidelines of the local ethical committee for in vivo experimentation.

### 4.2. Analysis of Serum Triacylglycerides, Cholesterol, Aspartate Aminotransferase, and Alanine Aminotransferase

Before sacrifice, blood samples were collected from abdominal aorta. Serum was separated by centrifugation (2000× *g* for 20 min) and tested for triacylglycerides (TAGs), cholesterol (CH), aspartate aminotransferase (AST), and alanine aminotransferase (ALT). All data were expressed as the mean ± SD of 3 to 5 mice per group and compared using one-way ANOVA.

### 4.3. Histological Analyses

Immediately after sacrifice, liver, heart, and kidney were weighted, and sections were fixed in 10% buffered formalin and processed for histological analysis (H&E) or immunohistochemistry (IHC). The remaining tissues were snap-frozen in melting isobutyl alcohol or in liquid nitrogen and stored at −80 °C until use.

To visualize the hepatic neutral lipid content, isobutyl alcohol-frozen liver sections were stained with Oil Red O (ORO, Sigma Aldrich, St. Louis, MO, USA) for 15 min, rinsed with 60% isopropanol, and stained with Mayer hematoxylin (Sigma Aldrich). The ORO staining positive area for each sample was quantified by using ImageJ analysis software (National Institute of Mental Health, Bethesda, MD, USA) [39,40]. The results were expressed as the mean ± SD of 3 to 5 mice per group.

### 4.4. Determination of Hepatocyte Proliferation

To evaluate cell proliferation following HFD-induced hepatocyte injury, BrdU (1 mg⋅kg^−1^ body weight) was administered to all mice from week 17 to 19. After sacrifice, mice tissues were fixed in 10% neutral-buffered formalin and embedded in paraffin. All tissues were cut at a thickness of 4 μm using Microm HM315 Rotary microtome (Rankin Biomedical Corporation, Holly, MI, USA), deparaffinized, and immunostained for BrdU to identify proliferating cells, as previously described [23]. Mouse monoclonal anti-BrdU antibody (Becton Dickinson, San Jose, CA, USA) and HRP anti-mouse antibody (Dako Corporation, Carpinteria, CA, USA) were used as primary and secondary antibody, respectively. Harris hematoxylin solution (Sigma Aldrich) was used to counterstain tissue sections. Pictures were taken using Zeiss Axio Lab.A1 (Carl Zeiss Microscopy, B, Oberkochen, Germany). Labeling index (LI) was expressed as the number of BrdU-positive hepatocyte nuclei/100 in ≥50 fields at 40× enlargement. The results were expressed as the mean ± SD of 3 to 5 mice per group.

### 4.5. Determination of Hepatic-Activated Fibrogenic Cells

An increased presence of hepatic cells positive for alpha-smooth-muscle actin (α-SMA) is associated with the progression of hepatic fibrosis in the liver. To determine whether 20-week-long HFD could induce fibrosis, and whether three-week-long treatment with MGL-3196 or TG68 could worsen or ameliorate the fibrosis stage, 4 μm-thick deparaffinized liver sections were immunostained for α-SMA, the most reliable marker of hepatic stellate cells activation. Mice treated with carbon tetrachloride (CCl_4_) were used as positive controls for the detection of α-SMA-positive cells. Mouse monoclonal anti-alpha smooth muscle actin antibody (Abcam, #ab7817, Cambridge, UK) and HRP anti-mouse antibody (Dako Corporation) were used as primary and secondary antibody, respectively. Harris hematoxylin solution (Sigma Aldrich) was used to counterstain tissue sections. Pictures were taken using Zeiss Axio Lab.A1 (Carl Zeiss Microscopy).

### 4.6. RNA Extraction and qRT-PCR

Total RNA was extracted from snap-frozen mice liver tissues using Qiazol Lysis Reagent (Qiagen, Hilden, Germany) followed by RNeasy extraction kit (Qiagen). Extracted RNA was reverse-transcribed using a High-Capacity cDNA Reverse Transcription kit with RNase inhibitor (Thermo Fisher Scientific, Waltham, MA, USA). RNA was quantified by NanoDrop ND1000 (Thermo Fisher Scientific), while RNA integrity was assessed by Agilent Bioanalyzer 2100 (Agilent, Santa Clara, CA, USA). Gene expression analysis was performed using TaqMan Gene expression Master Mix (Thermo Fisher Scientific) and the following specific TaqMan probes: *Dio1* #*Mm00839358_m1*, *Thrsp* #*Mm01273967_m1*, *Acox1* #*Mm00443578_g1*, *Srebf1* #*Mm00550338_m1*, *Fasn* #*Mm00662319_m1*, *Pnpla2* #*Mm00503040_m1*, *Cpt1* #*Mm00550448_m1*, *Acta2* #*Mm00725412_s1*, *Me1* #*Mm00782380_s1*, *Lgals3* #*Mm00802901_m1*, *Myh6* #*Mm00440359_m1*, *β-actin* #4*352933E* (Thermo Fisher Scientific). Each sample was run in triplicate, and all measurements were normalized to *β-actin*. Relative mRNA expression analysis for each gene was calculated by using the 2^−ΔΔCt^ method.

### 4.7. Statistical Analyses

All data are expressed as mean ± standard deviation (SD). Differences between groups were compared using unpaired two-tailed Student’s *t*-test and non-parametric Mann–Whitney U Test, or one-way ANOVA followed by Tukey *post hoc* analysis using the GraphPad Prism version 9.0.2 software for Mac OS (La Jolla, California, USA, www.graphpad.com, accessed on 30 November 2021). *p*-values were considered significant at *p* < 0.05.

## Data Availability

Not applicable.
